# Follow the sound of my violin: Granger causality reflects information flow in sound

**DOI:** 10.3389/fnhum.2022.982177

**Published:** 2022-11-03

**Authors:** Lucas Klein, Emily A. Wood, Dan Bosnyak, Laurel J. Trainor

**Affiliations:** ^1^McMaster Institute for Music and the Mind, McMaster University, Hamilton, ON, Canada; ^2^Department of Psychology, Neuroscience & Behaviour, McMaster University, Hamilton, ON, Canada; ^3^Rotman Research Institute, Baycrest Hospital, Toronto, ON, Canada

**Keywords:** Granger causality, cross-correlation, amplitude envelopes, information flow, music performance, expressivity, synchrony

## Abstract

Recent research into how musicians coordinate their expressive timing, phrasing, articulation, dynamics, and other stylistic characteristics during performances has highlighted the role of predictive processes, as musicians must anticipate how their partners will play in order to be together. Several studies have used information flow techniques such as Granger causality to show that upcoming movements of a musician can be predicted from immediate past movements of fellow musicians. Although musicians must move to play their instruments, a major goal of music making is to create a joint interpretation through the sounds they produce. Yet, information flow techniques have not been applied previously to examine the role that fellow musicians' sound output plays in these predictive processes and whether this changes as they learn to play together. In the present experiment, we asked professional violinists to play along with recordings of two folk pieces, each eight times in succession, and compared the amplitude envelopes of their performances with those of the recordings using Granger causality to measure information flow and cross-correlation to measure similarity and synchronization. In line with our hypotheses, our measure of information flow was higher from the recordings to the performances than vice versa, and decreased as the violinists became more familiar with the recordings over trials. This decline in information flow is consistent with a gradual shift from relying on auditory cues to predict the recording to relying on an internally-based (learned) model built through repetition. There was also evidence that violinists became more synchronized with the recordings over trials. These results shed light on the planning and learning processes involved in the aligning of expressive intentions in group music performance and lay the groundwork for the application of Granger causality to investigate information flow through sound in more complex musical interactions.

## 1. Introduction

Coordination through social interaction, including the ability to coordinate movements with others in time and space (joint action) underpins many complex, cooperative tasks that are unfeasible for individuals acting alone (Sebanz et al., 2006). In humans, action synchronization can increase cooperation, trust and prosocial behavior (Hove and Risen, 2009; Marsh et al., 2009), even in infants (Cirelli et al., 2014). Acting in concert with one another has enabled many cultural and technological advancements that would have been impossible otherwise (Cosmides et al., 2010; Tomasello, 2014). Benefits conferred by successful coordination in the challenges of everyday life may explain how these abilities developed in humans, and why group activities such as team sports and ensemble music performance are ubiquitous across cultures and throughout history (Axelrod and Hamilton, 1981; Morley, 2002).

Group music performance is a unique form of social interaction in that it requires a high degree of coordination among individuals. Typically, ensemble musicians share (or evolve) a common aesthetic goal that requires communicating the musical structure and their expressive intentions to each other and to their audience (Keller, 2014). Often this is achieved through the coordination of multiple distinct musical parts played simultaneously. At least in the Western classical musical tradition, to achieve a cohesive musical product, musicians must continually agree on or negotiate a set of shared expressive intentions and coordinate their actions to communicate them. This requires an awareness on the part of each performer of how and what their co-performers are playing—they must agree on or negotiate (often non-verbally) the character of the music they wish to convey because no individual part is heard in isolation. In the Western tradition, a collective musical expression from multiple separate parts requires temporal alignment of both note onsets (the precise timing of when notes are to be played), and the expressivity or character with which a piece is played. Expert musicians exert a large degree of control over the sound of any one note or sequence of notes by making continual adjustments to how they play their instruments. On the level of the performance, this translates to phrasing and articulation, dynamics (intensity), expressive timing, the use of vibratos, caesuras, and fermatas, and many other stylistic features of a performance—all of which unfold over time but are not strictly aligned with the musical beat.

While the musical context, as well as complexity, familiarity, and the musicians' expertise, can affect how alignment of expressive intentions takes place (Keller, 2014), it fundamentally entails a type of non-verbal communication in which musicians sense each others' actions to infer their musical intentions. By analogy, when two friends move a couch together, they communicate when and where they intend to move it through a *haptic* channel (the couch itself). Musicians, on the other hand, communicate their intentions largely through auditory and visual channels by watching and listening to each other play (even if the notes themselves are predetermined). Musicians' ancillary body movements—those not related to the *functional* purpose of producing the notes themselves—can serve a *communicative* purpose, signaling visually how and when to play (Wanderley et al., 2005; Pezzulo et al., 2019). Gestural motion, eye-gaze, facial expressions, head motion, and body sway (movements not directly related to producing sound) have all been shown to play communicative and expressive roles in group performances (Wanderley et al., 2005; Davidson, 2012; Chang et al., 2017; Bishop et al., 2019a). One study of qualitative observations from video recordings found that a piano duo increased their eye-contact and gestural cues (hand movement and torso-swaying) during “musically important” periods of performances (Williamon and Davidson, 2002), and another showed that gestural cues in leading violinists and pianists indicated tempo and beat positions (Bishop and Goebl, 2018). Noticing and interpreting micro-variations in the sounds and movements produced by fellow musicians—and indeed acting to produce them—is necessary for ensemble members to coordinate their actions in the pursuit of their shared aesthetic goals.

This automatic interpretation of sensory signals from other performers makes ensemble performance a valuable context within which to study non-verbal communication and the group coordination that it makes possible (D'Ausilio et al., 2015). Ensemble musicians exchange sensory information within a set of physical constraints (those inherent to playing an instrument) and rule-based constraints of the musical conventions, style, and genre. These conditions allow for tightly controlled experiments within naturalistic settings that are easily repeatable and can be adapted for different size groups, from duets, quartets, and jazz bands to full symphony orchestras.

While large orchestras usually benefit from an external timekeeper (a conductor who communicates tempo, dynamics, and other stylistic aspects of a performance to all the musicians simultaneously), smaller ensembles such as string quartets function as self-managed teams wherein each member contributes and group cohesion is particularly important for success (Murnighan and Conlon, 1991; Cohen et al., 1996; Davidson and Good, 2002; Luck and Toiviainen, 2006). Although the four members in a standard string quartet (first violin, second violin, viola, and cello) generally occupy different roles (the first violin most frequently functions as the “leader”), they are all responsible for arriving at a shared interpretation of the score and coordinating their playing to convey it. This means not only negotiating the expressive characteristics of the music, but also aligning their notes in time. One way musicians can accomplish this is by setting up explicit leader-follower relationships. This places more responsibility on one of the performers at any one time to set the tempo and convey dynamic changes and other expressive characteristics. Indeed, when a member of a string quartet is assigned as leader, they tend to exaggerate their bow movements (Timmers et al., 2014) and, compared to followers, leaders' head movement acceleration better indicates the beat (Bishop and Goebl, 2018). Even brain activity differs between leaders and followers (Novembre et al., 2014; Vanzella et al., 2019). To some extent, the other musicians may react to the sounds and movements of the leader once they hear and see them, but because it takes time to plan motor movements, relying on a reactive strategy such as this would leave the musicians out of sync with the leader. A more effective strategy for playing synchronously would be to anticipate fellow musicians' actions and sounds before they occur, and to plan their own in accordance with how they predict their partners will play (Sebanz and Knoblich, 2009; Moore and Chen, 2010).

Leadership dynamics in string quartets and orchestras have been studied in the context of how musicians use the sway of each other's bodies to predict how they will move next using Granger causality (e.g., D'Ausilio et al., 2012; Chang et al., 2017; Hilt et al., 2019). Granger causality (GC) is a measure of directed functional connectivity that quantifies how well information contained within the past of one time series (e.g., body sway of one musician) helps predict the current value of another (e.g., body sway of a second musician) based on vector-autoregressive (VAR) modeling (Granger, 1969). One time series is said to “Granger-cause” another if its history helps predict the time series' current value above and beyond prediction based on that time series' own history. When this is the case, information is said to flow from one time series to another. While such a measure cannot rule out the existence of a hidden variable that is driving the “causality,” it is a useful tool for examining prediction, and is sometimes referred to as “Granger prediction” to avoid the implication that causality is necessarily involved (Cohen, 2014). Nevertheless, the ability of one time series to predict another implies some form of communication must have taken place, an area of inquiry to which GC has been successfully applied. GC has proven to be a useful tool for quantifying the communicative capacities of body motion among skilled musicians in several studies of string quartets. Badino et al. (2014) measured GC between head movement time series of all four members of a string quartet while introducing perturbations known only to the leader. The total inter-group communication, as measured using GC between all the members, increased during periods following the perturbations, and when playing more complex pieces. Chang et al. (2017) found that the body sway of secretly assigned leaders in a string quartet influenced that of followers more than vice versa, and more than followers influenced each other. Assigning different members of the quartet as leader changed their relative predictive influences on the other members.

The cognitive processes that underlie inferring and predicting a partner's goals and actions have been proposed to stem from “common coding” (see Prinz, 1990), which hypothesizes a functional relationship between the perceptual and motor systems (Prinz, 1997; Schütz-Bosbach and Prinz, 2007). Perceiving another's actions can affect the performance of one's own related actions. For instance, reaction times of participants making perceptual judgments in a dual-choice button-press task show a similar compatibility effect compared to a those for a go-nogo task in the presence of a partner making complementary button presses, suggesting that one's own actions and the complementary actions of another are similarly represented (Sebanz et al., 2003). In a study comparing solo and duo conditions in a dot-stimulus tracking task, Knoblich and Jordan (2003) found that the presence of auditory cues regarding a partner's actions enhanced group performance. The ability for musicians to continually arrive at shared musical goals may rest on such perception-action links whereby partners simulate each other's intentions (Knoblich and Sebanz, 2008; Keller et al., 2014). In essence, they rely on or evolve shared musical (sound-based) goals to develop a dynamic internal representation of their partner's actions that can be used to make predictions about their partners' musical goals (Sebanz and Knoblich, 2009).

The ability to make predictions based on sound alone has been shown to be important for coordinating precisely during performances. One study of piano duos found that the ability to imagine sounds produced by others before hearing them (anticipatory auditory imagery) correlated with body movement coordination quality measured using motion capture (Keller and Appel, 2010). Another study found that individual differences in auditory prediction abilities modulated accuracy in an interpersonal sensorimotor synchronization task involving tapping with a partner (Pecenka and Keller, 2011).

Most examinations of sound-based prediction and synchronization in ensembles have focused on how musicians precisely synchronize the timing of their notes (e.g., by measuring the interval between note onsets of two or more musicians). This approach is particularly well-suited to percussive instruments such as pianos for which note-timing information is easily accessible (e.g., *via* MIDI recordings). Note onset asynchronies between two pianists have been used to show that decreasing auditory feedback decreases interpersonal synchronization (Goebl and Palmer, 2009), and that familiarity with a co-performer's part affects synchronization on short time-scales (keystrokes) and long time-scales (body movements) differently (Ragert et al., 2013). MacRitchie et al. (2018) used mean absolute asynchronies between pianists' notes to tease apart how incongruencies between individual and joint goals differentially affect synchrony. However, with non-percussive instruments, musicians can continuously vary the sound of their instrument, including on time scales shorter than single notes. For example, in stringed instruments, pitch and dynamics (loudness) can change markedly within the duration of one bow stroke, and wind instruments can expressively vary sound characteristics including timbre and vibrato on a continuous basis. Despite this, few studies have examined how musicians make predictions based on the continuous sounds produced by their fellow musicians, and fewer still have used measures of information flow. Granger causality (GC) (Chang et al., 2017) and mutual information (Ragert et al., 2013) have been applied to body movements, but information flow between the continuous sounds of performing musicians remains essentially unstudied. To our knowledge, GC has not been used to date to study information flow between musicians' musical sound outputs, although it has been used to analyze influences of acoustic properties on perceptual responses (Dean and Bailes, 2010; Bailes and Dean, 2012).

One goal of the present study was to examine whether musicians anticipate what a partner will play solely based on the immediate past of the sounds produced by that partner, by applying GC to the time series of the musicians' audio outputs. At least one study suggests indirectly that this may be the case. Examining GC between the body sway movements of the musicians in a string quartet, Chang et al. (2017) found that even when the musicians could not see each other, the body sway of one musician influenced the body sway of the others (and information flow from the body sway of leaders to followers was greater than vice versa). Given that they could not see each other, body sway could not have been the direct communicative cue. Rather, body sway likely reflects musicians' planning processes related to the sounds they are producing, similarly to how hand gestures reflect planning of speech (Graham and Heywood, 1975; Morsella and Krauss, 2004), and the musicians' sounds themselves contained cues for predicting how each other planned to play in the future. If so, these cues must be present in audio recordings of musical performances. We based our analyses on the audio *amplitude envelope*, a time series consisting of a smooth curve that tracks variations in amplitude (intensity or loudness) over time. We focused on the amplitude envelope because it encodes time-variant acoustic properties of the sound signal.

A second goal of the present study was to examine how auditory-based prediction processes change as a musician becomes familiar with (i.e., learns) to mimic another musician. In this regard, a prominent framework of social interaction involves the idea that co-actors form (learn) *internal models* that simulate the link between motor commands and their sensory consequences across the co-actors (Wolpert et al., 2003). In this framework, when musicians share a common forward (causal) internal model, this allows them to predict each other's actions and their sensory consequences (sounds), enabling them to play synchronously (Heggli et al., 2019). When they accomplish this, the sounds they produce coordinate, and they can arrive at a joint musical expression. According to this framework, we expected that in learning to play a piece together, musicians would initially rely on predictive cues from the movements and/or sounds of the other musicians, but that with practice, the musicians would come to a common musical interpretation, thus forming common internal models. They would then rely less on direct predictive cues through seeing or hearing each others' movements or sounds. Indeed, there is evidence that average GC based on body sway decreases as musicians learn to play a piece together (Wood et al., 2022), and that rehearsal improves movement coordination among piano and clarinet duos (Bishop et al., 2019b). In the present study, we measured Granger causality when sound was the only communicative cue present.

To control the auditory cues present during learning, in the present study, we examined how violinists learn to play with prerecorded pieces containing large amounts of expressive liberty by having them play with each prerecorded violin piece eight times in succession. In this scenario, the only information available for the creation of an internal model of the expression is the sound recording. Our main hypothesis was that the violinists would initially rely mainly on predictive processes based on what they were hearing in the recording—for example, predicting the dynamics and expressive timing of the prerecorded violinist based on their immediate past dynamics and expressive timing as the piece unfolded in time. Over successive playing with the recording, if violinists formed improved internal models of the expressive interpretation of the music on the recording, we would expect them to rely more and more on predictions based on their internal models and less and less on predictions based on the sound itself. We therefore expected that an analysis of Granger causality would show a decrease in information flow from the recording audio to the violinists' audio across repetitions as the violinists learned how the musician on the recording interpreted the piece.

In addition to information flow, we measured the similarity of amplitude envelopes of the violinists' performances and the recordings across pieces and repetitions using cross correlation (CC). CC measures the correlation between two time series across time-delayed (“lagged”) copies of one another within a range of positive and negative lags. The CC measure is taken to be the largest CC value across the range of lags. To some degree, CC and GC are complementary measures because information can only flow between time series that are sufficiently dissimilar from one another; Granger causality between two identical time series will necessarily be zero because the second time series cannot add additional information that is not already represented in the first time series. However, both GC and CC are directional measures to an extent. For example, information flow would be expected to be higher from the recording to the violinist than vice versa as the violinist should not be able to influence the recording. Similarly, the sign of the lag at which the largest CC value occurs suggests the temporal precedence between the two time series. While the calculation of CC does not rely on statistical prediction like GC, if one process (e.g., the recording) influences another (e.g., the violinist's performance), we would expect a high CC value to occur when the performance lags behind the recording, and not vice versa. It should be noted that CC does not measure how synchronous the time series are but rather how similar they are. One measure of synchrony is to measure CC with a zero lag (when the two time series are positioned together in time).

In sum, first, we expected GC values to indicate higher information flow from the recording to the performances than vice versa across all trials. Second, because the recording was initially unfamiliar, we expected information flow from the recording to the performances to decrease over successive trials as the violinists became familiar with what they were attempting to match; the more information about the piece they could rely on obtaining from memory (an internal model), the less information they should need from hearing the recording while playing. Third, we expected CC similarity to be highest at lags for which the recording preceded the performances and, fourth, for both similarity and synchrony measures between the recording and the performances to increase across trials as they memorized the expression of the recording and learned to match it more effectively.

## 2. Materials and methods

### 2.1. Participants

We recruited nine professional violinists (five female) from around the Hamilton, ON area and one from Arizona, USA, willing to participate in a remote study and record themselves at home. All participants reported that they performed in a professional capacity in orchestras or chamber music groups, and they all had obtained professional music-related academic degrees (e.g., Bachelor of Music Performance, Master's Degree in Music). Most participants also reported playing in solo performances, and some had their own teaching practices or recording studios. They had an average of 36 years of musical experience (*SD* = 9.6) on their primary instrument (violin for all except one whose primary instrument was viola) and practiced for an average of 18 h per week (*SD* = 13.9) at the time of data collection.

### 2.2. Stimuli

We sourced recordings of the popular folk tunes *Danny Boy* (Piece 1) and *In The Garden* (Piece 2) from the website www.violinsolos.com, played on solo violin, with accompanying sheet music. We looked for expressive performances so that there was some unpredictability; the performer loosely followed the sheet music, while incorporating tempo shifts, dynamic changes, caesuras, and fermatas. *Danny Boy* was chosen for the familiarity of its melody (all the participants had heard the song before), and relative simplicity. This rendition was played at ~55 beats per minute in 4/4 time (with one beat equal to one quarter note) in the key of F-major. We chose the second piece to be less familiar (no participants reported having heard the song before) and played at a faster tempo but in a similar expressive way. *In The Garden* was performed at ~130 beats per minute in 6/8 time (with one beat equal to one eighth note) in the key of B-flat major. This piece was slightly more complex in including double-stops. For both pieces, we removed all markings from the sheet music, including dynamics and bowing, so that it contained only the clef, key signature, time signature, barlines, and notes (see Supplementary materials for the sheet music).

**Figure 1:**
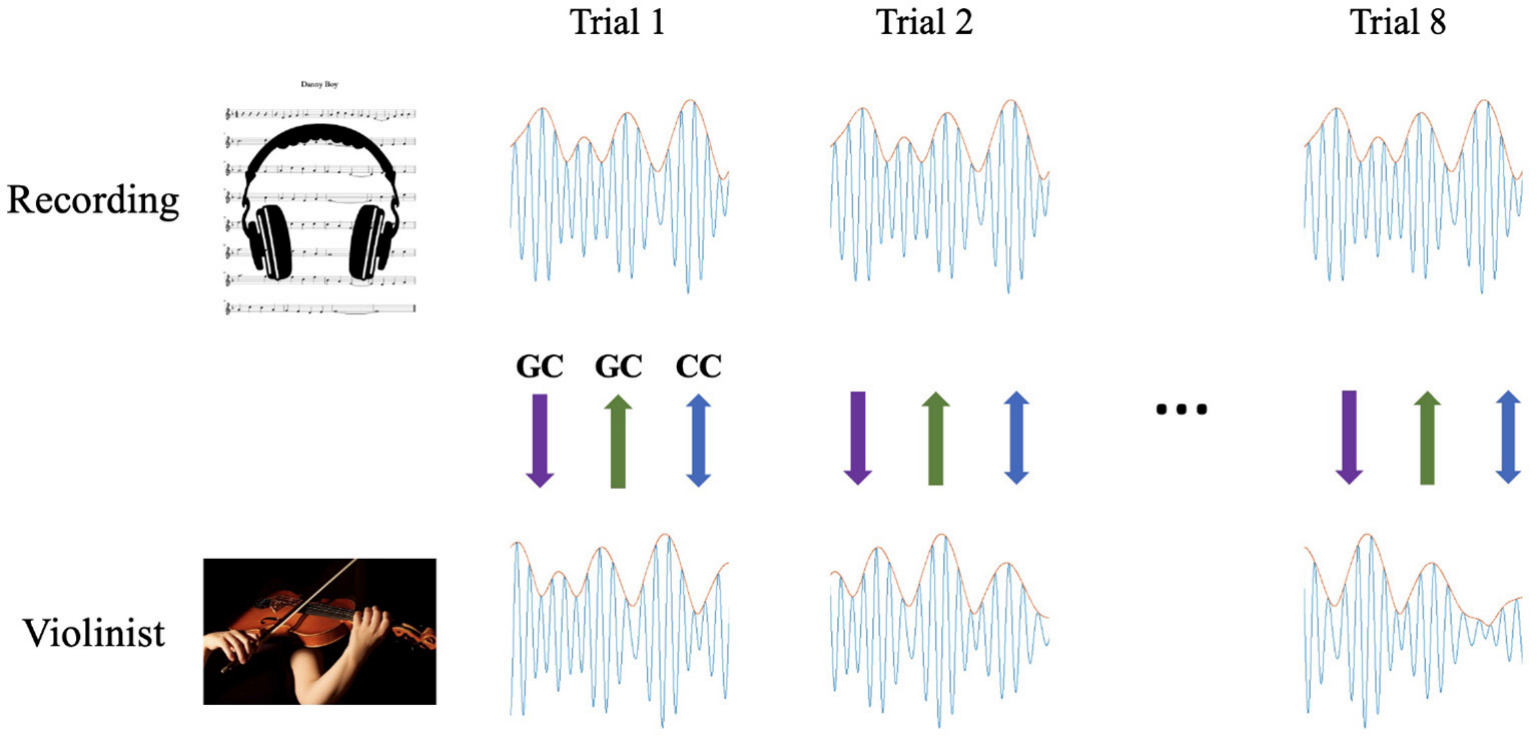
Experimental design and procedure. The violinists listened to the recordings through headphones while looking at the sheet music **(top)** and recorded themselves playing along with them **(bottom)** across eight successive trials. We then calculated Granger causality in both directions (from the recordings to the performances, shown in purple, and from the performances to the recordings, shown in green) and cross-correlation (for similarity and for synchrony, shown in blue) for each trial separately.

The stimulus recordings were created using *Studio One 3* (Presonus Audio Electronics, 2020). The first ~90 s of each song was extracted. For each song, eight consecutive identical trials were presented, each consisting of the 90-s excerpt. Voice instructions between each trial indicated that there would be a 15-s period of silence before the pickup clicks for the next trial began. *Danny Boy* included five clicks (one full measure plus the first beat of the measure in which they started playing) preceding the initial three pickup quarter notes of the piece, and *In The Garden* included eight clicks (six eighth note clicks to represent one full measure plus two clicks each representing dotted quarter notes in the measure in which they started playing) preceding the initial pickup eighth note of the piece. The pickup clicks indicated only the starting tempo of the pieces. The entire data collection process for each piece lasted ~15 min. Full stimulus tracks for both pieces (mixed down as monophonic 16-bit WAV files at 44.1 kHz sampling rate) and accompanying sheet music were sent to violinists in advance, with instructions not to listen to them or play them prior to conducting the experiment. In addition, we created a “practice version” of the stimulus using a different solo violin recording, *Amazing Grace*, performed by the same musician and presented during practice trials in the same way as the other pieces were presented in the experimental trials. We used the practice trials to test participants' recording setups and to familiarize them with the procedure. All the violinists played *Danny Boy* before playing *In The Garden*. See Figure 1 for a depiction of the experimental setup.

### 2.3. Data collection

Seven of the nine total violinists recorded themselves playing both pieces, one additional violinist played only *Danny Boy* (Piece 1), and another additional violinist played only *In The Garden* so that each piece was played by eight violinists in total. Data were collected at violinists' homes with their own recording hardware and software. Most recorded their sound using a desktop microphone connected to a USB audio interface while simultaneously listening to the stimulus violin track. Because Granger Causality requires the time series of the stimulus track and the participants' recording to be temporally aligned, it was crucial to synchronize the recording of each violinist's performance with the presented audio. The violinists imported the stimulus WAV files we sent them into their recording software (Audacity, Logic Pro, GarageBand, Studio One, Reaper, or Avid ProTools) as one track and made a second track to record themselves. They then recorded themselves playing into the second track (at 44.1 kHz sampling rate) while listening to the first track through headphones. In most cases, participants used wired, non-noise-canceling headphones (open-back headphones or in-ear buds) so they could also hear the sound of their own instrument over the recording. They recorded all eight trials in succession in one take (see Figure 1). They then mixed down both tracks as separate 16-bit WAV files and sent them back to us. Using the same set of time markers for each piece, we cut each participant's performance and recording files into separate tracks for each trial (the recording for each trial began at the beginning of the participants' playing and excluded the pickup clicks). Theoretically, each stimulus track should be exactly the same, but we analyzed versions exported directly from the violinists' own recording software to account for differences in track levels and other software-specific settings. This resulted in 16 WAV files for each of eight participants for each of two pieces.

### 2.4. Data analysis

#### 2.4.1. Amplitude envelopes

Waveforms were extracted from the WAV files as time series using the SciPy package for Python. They were rectified (absolute value taken) and filtered twice, once forward and once backward, using a 3rd order Butterworth IIR filter with a critical (cutoff) frequency of 11.025 kHz (half the Nyquist frequency, with the 44.1 kHz sampling rate), resulting in arrays that represented the amplitude envelope time series with the same length as the waveforms. The arrays were saved as text files and then downsampled to ~8 Hz (5,513 points) by averaging the time points within consecutive, non-overlapping 125-ms windows.

#### 2.4.2. Granger causality

We calculated the magnitude of Granger causality (GC) from the amplitude envelope time series of the recordings to the amplitude envelope time series of the performances—and vice versa—for each participant and each trial following the procedure implemented in the Multivariate Granger Causality (MVGC) Toolbox for MATLAB (Barnett and Seth, 2014). All time series met the assumption of stationarity required for GC. An optimal model order (the number of past points in the time series included in the model) was chosen for each trial for each participant using the Akaike information criterion. Then, for each participant for each piece, the maximum model order out of their eight trials was used to calculate GC values for all eight trials. In other words, model orders were specific to each participant and piece, but within a participant, the same model order was used for all trials of the same piece. The average model order for participants when playing *Danny Boy* was 6.63 (*SD* = 0.52), which corresponded to 0.829 s. The average model order for participants when playing *In The Garden* was 8.63 (*SD* = 1.41), which corresponded to 1.079 s.

#### 2.4.3. Cross-correlation

To measure the similarity between the sounds of the violinists' performances and the recording they followed, we calculated cross-correlations (CC) between the amplitude envelope time series of the recordings and the performances for each trial for each participant. CC coefficients were calculated across the entire waveforms for each trial for lags between −10 and 10 points (10 points amounts to 1.25 s; approximately the duration of three eighth notes in each piece, corresponding to about 1.5 beats for *Danny Boy* and three beats for *In The Garden*). The maximum of the coefficients across all time lags was taken as the CC value for each trial, resulting in one cross-correlation coefficient for each trial for each participant. Because this CC measure evaluates similarity but not necessarily synchrony between the recording and performer (maximum correlations could have occurred at any lag within our range), we repeated the CC analysis while confining the time lag to zero and used this as a measure of phase alignment or synchrony between the two amplitude envelopes.

The lag at which the maximum correlation occurred (optimal lag) in each trial indicates the time delay between the two time series that produced the highest degree of similarity. A positive optimal lag indicates that the time series were most similar when the recording preceded the performance, and vice versa for a negative optimal lag. An optimal lag of zero indicates no temporal precedence between the time series (i.e., synchrony).

## 3. Results

### 3.1. Information flow direction

**Figure 2:**
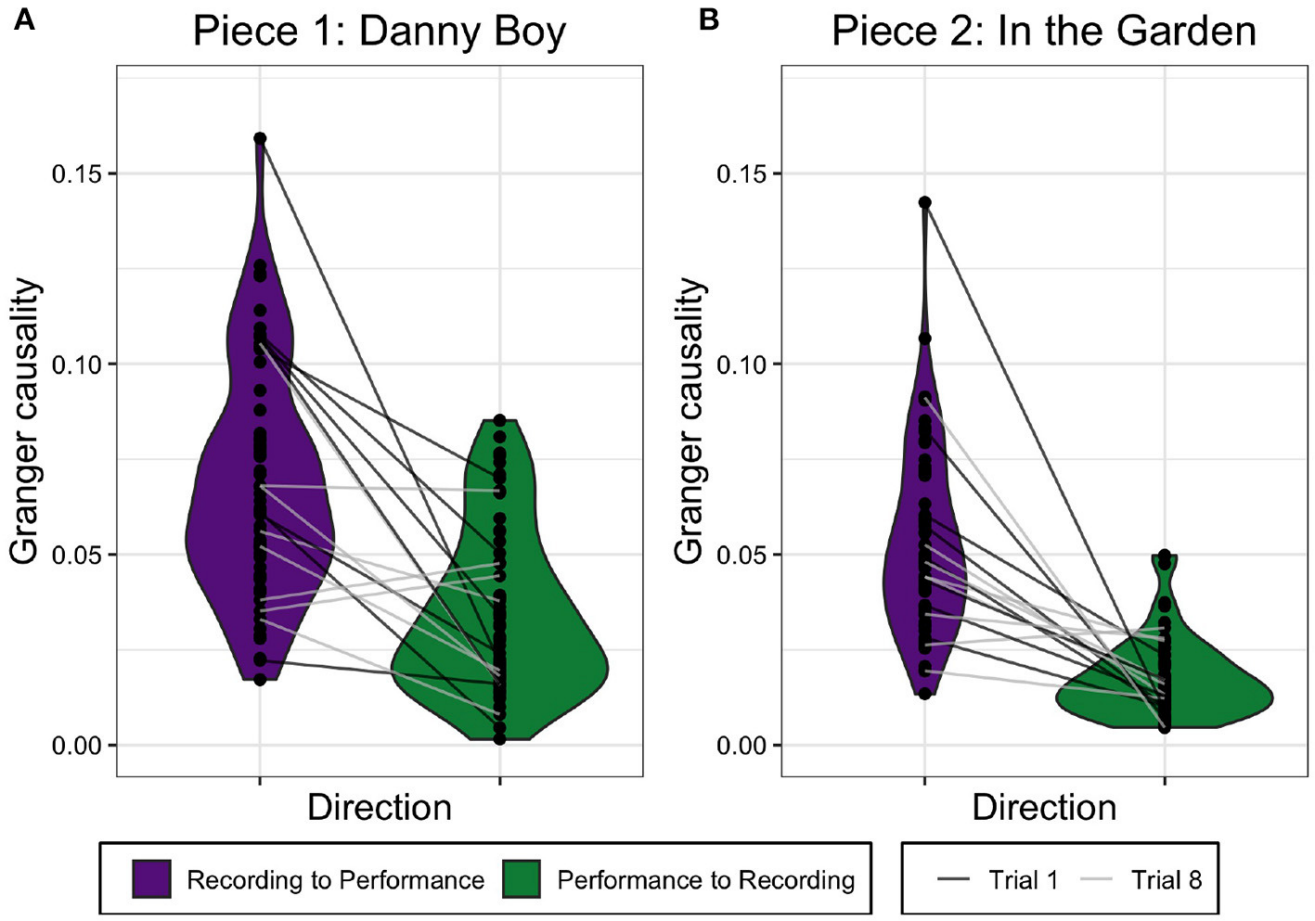
Granger causality values for both directions, recording to performance and performance to recording, for *Danny Boy* **(A)** and *In The Garden* **(B)**. The lines show how each participant's GC values changed between the two directions for Trial 1 and Trial 8.

We compared the GC values in the two directions (performance to recording; recording to performance) within-subjects for each piece separately (see Figure 2). GC values from the recording to the performance were larger than from the performance to the recording across all trials for Piece 1 (*Danny Boy*), *t*(63) = 7.038, *p* < 0.001, and for Piece 2 (*In The Garden*), *t*(63) = 9.659, *p* < 0.001. These results are as expected; because the recording was fixed, information was expected to flow from the recording to the performance rather than the reverse. This establishes that information flow between the musical output of two violinists can be meaningfully measured.

### 3.2. Changes in information flow across trials

**Figure 3:**
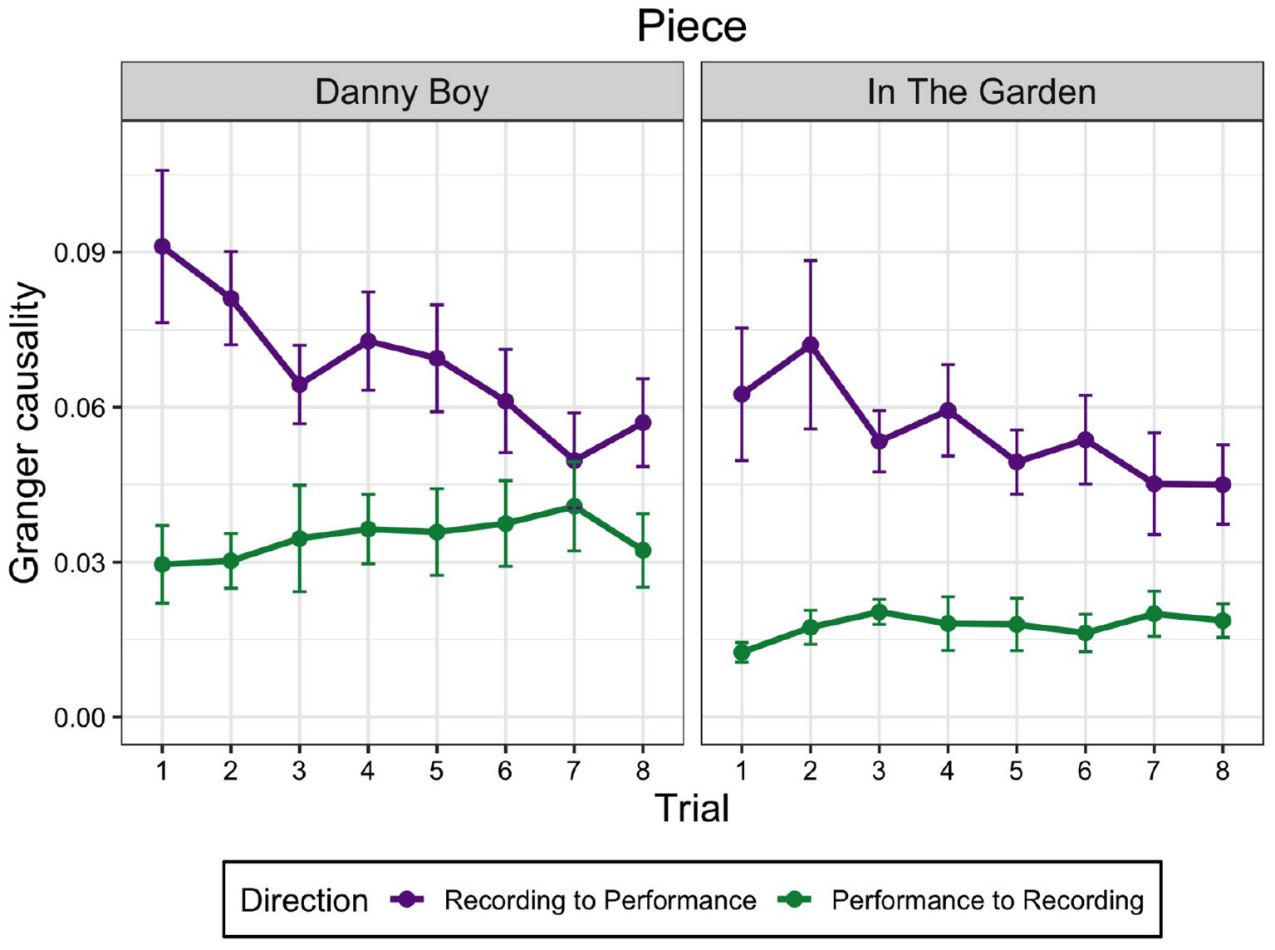
Granger causality (GC) values in both directions for each piece separately across the eight trials. Error bars represent standard error.

The violinists became more familiar with the recordings after each successive trial. To test the effect of trial on information flow, we modeled GC (only from the recording to the performance) as an outcome variable in a linear mixed effects model using the “lme4” package in R version 4.2.1 for each piece separately, as the pieces differed in tempo, note density and other structural features. The maximum likelihood approach uses ANOVA to compare full and reduced mixed models. Our full model included trial (eight trials) as a fixed effect, and participant (eight participants) as a random effect. The reduced model was identical except that it excluded the fixed effect of trial. The coefficient estimates of trial for Piece 1 were β = −0.005 with semi-partial *R*^2^ = 0.225 and, for Piece 2, β = −0.003 with semi-partial *R*^2^ = 0.199. Both were statistically significant (*p* < 0.001). This indicates that GC decreased significantly across trials from the recording to the performance (see Figure 3).

To examine the nature of the decrease in GC values across trials, we ran a set of linear contrasts (comparisons) including all eight levels of trial (as a quantitative ordered factor). This revealed a significant decreasing linear trend of GC for Piece 1, *F*(1, 56) = 9.819, *p* = 0.003, and for Piece 2, *F* (1, 56) = 4.141, *p* = 0.047, indicating a linear decrease over trials in both cases as hypothesized.

### 3.3. Changes in similarity and synchrony across trials

**Figure 4:**
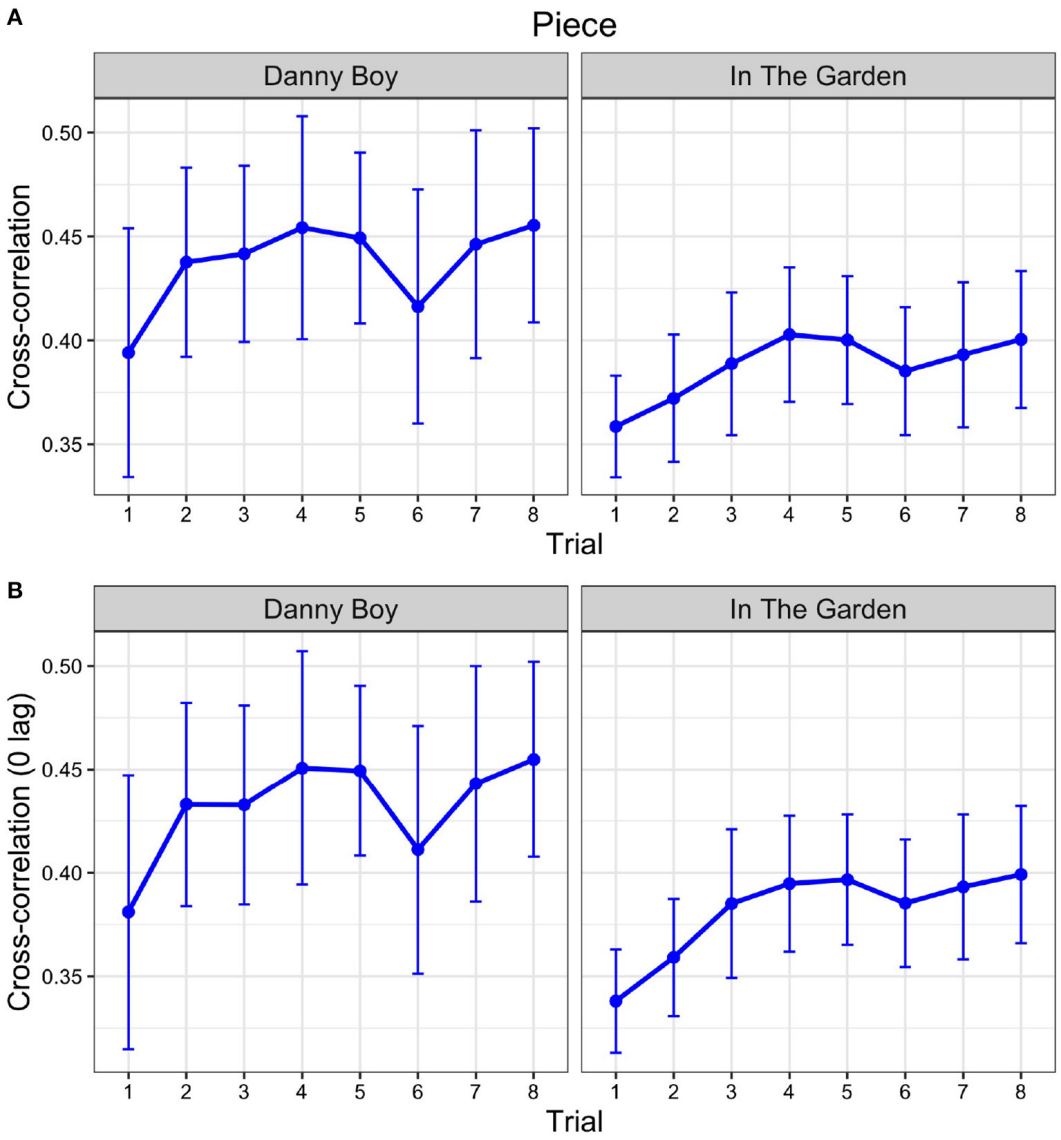
Cross-correlation (CC) values for each piece separately across the eight trials. **(A)** Similarity, calculated using the maximum CC value on each trial for lags between −1.25 and +1.25 s. **(B)** Synchrony, calculated using a lag of 0 s.

We modeled the CC coefficients as fixed effects of trial and random effects of participant, using the same full and reduced models as for GC, except with the maximum CC coefficient for each trial as the outcome variable. Coefficient estimates of trial were significant for Piece 1 (β = 0.005, semi-partial *R*^2^ = 0.069, *p* = 0.046), and trending significant for Piece 2 (β = 0.005, semi-partial *R*^2^ = 0.062, *p* = 0.057). Linear contrasts identical to those for GC were also run on the CC values, but neither piece produced a significant linear trend [for Piece 1, *F*(1, 56) = 0.357, *p* = 0.553, and for Piece 2, *F*(1, 56) = 0.887, *p* = 0.350; see Figure 4A].

Our measure of maximum CC coefficient for similarity, above, (using a lag window of ±1.25 s) does not necessarily capture phase alignment or synchrony between the two times series as the maximum CC value may occur when the two time series are lagged with respect to each other. To examine synchrony, we restricted the lag to 0 and re-ran the same CC analysis. The linear mixed model estimate of trial was significant for the Piece 1 (β = 0.006, semi-partial *R*^2^ = 0.087, *p* = 0.024) and for Piece 2 (β = 0.007, semi-partial *R*^2^ = 0.146, *p* = 0.003). As for the similarity analysis, neither piece exhibited a significant linear trend with 0 lag [for Piece 1, *F*(1, 56) = 0.519, *p* = 0.474 and for Piece 2, *F*(1, 56) = 2.135, *p* = 0.150; see Figure 4B].

There were no significant differences across trials for what lags produced the maximum cross-correlations (optimal lags). Importantly, average optimal lags were almost all positive or zero (98.5%) for both pieces for all trials, indicating that similarity was greatest at a lag where the recording preceded the performance, or when the two were aligned, and never when the performance preceded the recording.

## 4. Discussion

A strong theoretical position states that social coordination in general and playing music with others in particular rely on predictive mechanisms because motor movements and communicative messages require time to plan (Knoblich et al., 2011; Keller, 2014; Keller et al., 2014; Dobson and Gaunt, 2015). Thus, reacting to the actions or sound output of others rather than anticipating them will make coordination with others difficult and synchronization inaccurate. While most previous studies in naturalistic musical contexts have focused on actions such as musicians' body sway to examine how musicians anticipate how each other will move in order to adjust the timing of their own movements (Goebl and Palmer, 2009; Glowinski et al., 2012, 2013; Ragert et al., 2013; Chang et al., 2017, 2019; Colley et al., 2018, 2020; Hilt et al., 2019; Wood et al., 2022), here we focused on the musical sound output itself to examine whether a musician can predict how another musician will play next based solely on sound. Previous research examining musicians' sound output in joint performance tasks has focused primarily on measures of synchrony, either between MIDI note onsets or tapping timing (e.g., Repp and Keller, 2008; Goebl and Palmer, 2009; Keller and Appel, 2010; Repp and Su, 2013), but none has directly measured information flow in the continuous sounds of performing musicians. Using the context of a Western musician playing with a recording, we were able to control one musician (the recording) to be identical for all the performing musicians. Our GC analyses clearly showed that information flow was much higher from the recording to the musicians than vice versa, indicating that musicians can learn to predict how another musician will play next on the basis of the sounds they just produced.

A second question of interest concerns two possible sources of predictive information and how musicians learn through practice to create a shared expressive model of how to interpret a piece of music together. Here we examined this in the simplified and controlled situation where one musician was a sound recording that a second musician performed with, playing each piece eight times in succession. We deliberately chose two solo violin pieces that offer room for a large amount of expressive variability so that simply playing the correct notes would not be sufficient; the musicians playing with the recording needed to match the recording musician's interpretation. One source of predictive information is directly in the musical sounds of the recording musician, whereby the performing musician may be able to predict how the recording musician will play next based on how they just played. A second source of predictive information is based on prior knowledge about how the musician on the recording is likely to play the piece. Initially, the performing musicians only have prior knowledge of the recording based on their understanding of the Western musical genre and how they may have heard the piece or similar pieces performed in the past. However, through repeatedly hearing the recording across the eight trials, we would expect the performing musicians to improve their prior knowledge by building up an internal representation (or memory) of precisely how the recording musician played the piece. In turn, this should allow them to anticipate and synchronize more accurately with the recording and come to rely more on this alternative source of predictive information. Consistent with this interpretation, we found, as predicted, that GC values from the recording to the performing musicians decreased across the eight trials, suggesting that as they became more familiar with, that is, learned, the expressive rendition of the recording musician, they relied moment to moment less on predictive cues based on what the recording musician had just played and more on predictions based on their memory or internal model built from having heard this particular performance repeatedly.

Performances typically consist of interacting live musicians, involving non-verbal communication and prediction of how each other will play, and the refinement through experience of an internal model or knowledge of how the other will play. The use of generative models to explain “top-down” influences on perception rests in the predictive processing paradigm (a centuries old philosophical tradition; Swanson, 2016) that also supports the active inference framework (Friston and Kiebel, 2009; Vuust and Witek, 2014; Friston and Frith, 2015; Heggli et al., 2019). In this view, independent interacting agents infer the causes of sensory information, which includes that generated by the other agent, and act to minimize their uncertainty. One caveat of our study is that the recording was fixed, so only the performing musician was able to adapt their internal model. Still, our results are consistent with the active inference perspective in that information flowed from the recording to the musician rather than vice versa, and that the performing musician came to rely less on immediate inferences (i.e., GC decreased) through practice, consistent with the musician building a more accurate internal predictive model through repetition.

As the performing musicians came to rely less on predictive cues in the musical sounds on the recording and more on an internal model based on learning (or memory of) the interpretation of the recording musician, we expected that their performances would become more similar to that of the recording musician. Past studies have used a cross-correlation (CC) measure of the similarity between musicians' movements and note onsets (e.g., Goebl and Palmer, 2009; Wing et al., 2014; Colley et al., 2018, 2020; Bishop et al., 2019b) and tapping timing (e.g., Pecenka and Keller, 2011; Schultz and Palmer, 2019). In the present study examining the musical outputs of musicians, we expected CC to increase across trials as GC decreased. Indeed, a previous study from our lab observed this inverse pattern in the body sway times series of members of a string quartet (Wood et al., 2022), where GC decreased across trials while CC increased. In line with our predictions, we found that CC increased across trials for both pieces (trending significant for Piece 2); however, linear trends for increasing CC across trials were not significant (as they were for decreasing GC values), indicating that CC increased more at some points during learning than at others, perhaps reflecting in part that musicians may have fatigued towards the end of the eight repetitions.

Given that CC measures the maximum absolute correlation within a range of time lags between the recording musician and the performing musician (±1.25 s), this measure does not necessarily inform us about how synchronized or phase aligned the performing musicians were with the recording. To examine this, we ran a CC analysis using only a lag of zero, that is, we examined correlations between the recording and performer sound outputs when aligned in time. In this case, we found that CC increased significantly across trials for both pieces. In sum, we found evidence that the sound outputs of the recording and performing musicians became more synchronized over trials—indicating that the performing musicians' ability to synchronize or phase align with the recordings increased the more times they heard and played the piece.

Our measures of similarity and synchrony displayed an almost-identical pattern of increasing values over trials. This means that restricting the range of time lags over which the cross-correlation coefficients were calculated had little effect because in most cases the maximum absolute coefficient occurred close to a lag of zero. However, these optimal lags were almost always positive or zero (98.5% including both pieces). The positive sign observed in these values indicates that the maximal similarity of the two time series occurred when the recording preceded the performance. Although this does not necessarily reflect any causal interaction, one process that causes another would be expected to occur ahead of it in time.

In interpreting these results, however, it should be kept in mind that we performed our analyses on the amplitude envelope of the sound. While the amplitude envelope captures details of intensity changes over time, it misses many other aspects of musical expression, including pitch changes. For instance, two or more slurred notes can be played in succession with a single bow stroke, producing very little change in the overall intensity of the sound; nor will the amplitude envelope capture expressive micro-pitch changes, such as those associated with vibrato. Thus, while the amplitude envelope reflects many of the small continuous adjustments musicians make, it should be considered an imperfect, albeit useful, proxy for musical expression and some expressive characteristics present in our participants' performances were likely inaccessible to our analysis. It is possible that a frequency-based comparison between the two sound streams, such as spectral coherence, could prove fruitful. Expert ratings of performance synchrony could serve as another useful (although non-objective) measure.

Several of our results differed slightly across the two pieces. In particular, the increase in similarity was only trending significant for Piece 2. Although minor, given that all but one violinist in each group performed both pieces, these differences suggest structural aspects of the two specific pieces played a role in violinists' process of learning to match or synchronize with them. Both pieces were chosen for the high level of expressive freedom taken by the performer but had other important differences, related to both difficulty and familiarity. All performing musicians reported having heard and played *Danny Boy* previously but none had heard *In The Garden* before, which we expected given the general popularity of *Danny Boy* within the context of Western music. Regarding difficulty, *In The Garden* had a faster tempo (130 vs. 55 beats per minute), a higher note density (~1.70 vs. ~1.09 notes per second) and included double stops, consistent with some violinists anecdotally reporting finding it more challenging. Both of these factors might be expected to affect learning rates.

In naturalistic contexts, musicians likely use movements, sights and sound to predict how each other plan to play, and the extent of their reliance on different cues likely depends on the situation. For example, changes in body movement dynamics in joint performance have been shown to track changes in task demands. Motion capture data revealed that head movements of violinists become more regular and predictable when playing in a quartet vs. solo (Glowinski et al., 2013) and that gestures become more coordinated and smoother during periods of temporal instability as a group learns unfamiliar music (Bishop et al., 2019b). Research in this vein highlights how sensory signals arriving by auditory and visual modalities interact; when one type of information is unavailable or insufficient, another may become more important. Leaders in piano duos tend to lift their fingers higher and play less synchronously when they get less auditory feedback from partners, suggesting that the lack of information in the auditory domain increased the importance of visual cues (Goebl and Palmer, 2009). Bishop and Goebl (2015) showed that pianists played less synchronously with a video recording of a performance when they could only see but not hear the recording, but that in this condition synchrony increased during periods after long pauses, pointing to the importance of visual cues for synchronizing when a partner's performance is difficult to predict.

The performance task we asked musicians to undertake of matching their performance to a recording was not representative of how they would typically perform. However, this highly controlled experiment clearly establishes that information flow in musical sound output between musicians reflects the dynamics of interaction and learning. It is exciting in opening the door to understanding subtleties of how musicians jointly create complex musical performances at the sound level. It also opens a rich set of questions regarding how movements (such as body sway) and auditory perception interact to achieve joint musical goals. Immediate questions that need to be explored include measuring information flow between the sounds of live pairs of musicians as well as larger groups. We would expect to see mutual predictive influences between the sounds of live musicians playing together, effects of leadership whereby the sounds of leaders affect the sounds of followers more than vice versa, increases in information flow when a piece is played more expressively, and decreases in information flow as a piece is rehearsed and common joint internal models are formed. Extending to more than one live musician presents challenges in measuring each musician's sound output separately and would require either pickups or highly directional microphones that can record each instrument's audio separately with little residual bleeding between the recordings, or signal processing techniques, or some combination of these. While MIDI can get around this issue, examining the rich sound output of non-percussion instruments that create continuous sound, as in the present study, would be very informative. Having musicians perform in separate rooms would solve this issue and introduce the ability to compare seeing and non-seeing conditions.

The present study investigated information flow through sound in the context of Western music performance in which musicians played a pre-composed piece from a score. However, many other styles and genres of music exist both within and beyond Western music. For example, in improvisation, such as occurs in jazz ensembles or jam bands, musicians compose new music in real-time. The increased uncertainty that this engenders may make predictions of what fellow musicians will play on the basis of what they just played even more important than in non-improvised contexts. Across cultures, there are marked differences in scales used, rhythmic complexity, degree of polyphony, and whether precise synchrony is a goal or not. To determine whether information flow and synchrony can reveal coordination dynamics of joint music making universally, they will need to be measured in many contexts and across different cultures. We hope that the results of this study, conducted in a highly controlled setting, will lay the groundwork for future application of Granger Causality to musical sound coordination in more varied and ecologically valid performance contexts. We believe this approach could serve as a useful tool for investigating information flow between the sound outputs of live performing musicians, similar to what has been done for body sway.

## Data availability statement

The scripts used to analyze the dataset for this study can be found in the online repository at: https://github.com/trainorlab/violinfollowing. The datasets are available from the corresponding author upon request.

## Ethics statement

The studies involving human participants were reviewed and approved by McMaster Research Ethics Board, McMaster University. The patients/participants provided their written informed consent to participate in this study.

## Author contributions

DB, LK, LT, and EW contributed to the conception and design of the study. LK created the stimuli, acquired the data, and performed the data analysis with help from EW. LK wrote the first draft of the manuscript, with edits from LT. All authors contributed to the interpretation of the results and revised and approved the manuscript.

## Funding

This research was supported by grants from Social Science and Humanities Research Council of Canada (SSHRC) [SSHRC Insight Grant (435-2020-0442)], the Natural Sciences and Engineering Research Council of Canada (NSERC) [NSERC Discovery Grant (RGPIN-2019-05416)], the Canadian Institutes of Health Research (CIHR) [CIHR Project Grant (MOP 153130)] to LT, and (the Canadian Institute for Advanced Research and an NSERC-CREATE Complex Dynamics of Brain and Behavior fellowship) to LK.

## Conflict of interest

The authors declare that the research was conducted in the absence of any commercial or financial relationships that could be construed as a potential conflict of interest.

## Publisher's note

All claims expressed in this article are solely those of the authors and do not necessarily represent those of their affiliated organizations, or those of the publisher, the editors and the reviewers. Any product that may be evaluated in this article, or claim that may be made by its manufacturer, is not guaranteed or endorsed by the publisher.
