# Supplementary material for: Follow the sound of my violin: Granger causality reflects information flow in sound
Source: Front Hum Neurosci. 2022 Nov 3;16:982177. doi: 10.3389/fnhum.2022.982177 (PMC9671163; doi:10.3389/fnhum.2022.982177)
Supplement: Supplementary file 1 [file Data_Sheet_1.PDF]

frontiers

Supplementary Material

1 SUPPLEMENTARY DATA

The violin recordings used in the stimuli for this study were sourced from and are available at the website www.violinsolos.com; *Danny Boy* can be found here, and *In The Garden* can be found here.

2 SUPPLEMENTARY TABLES AND FIGURES

2.1 Figures

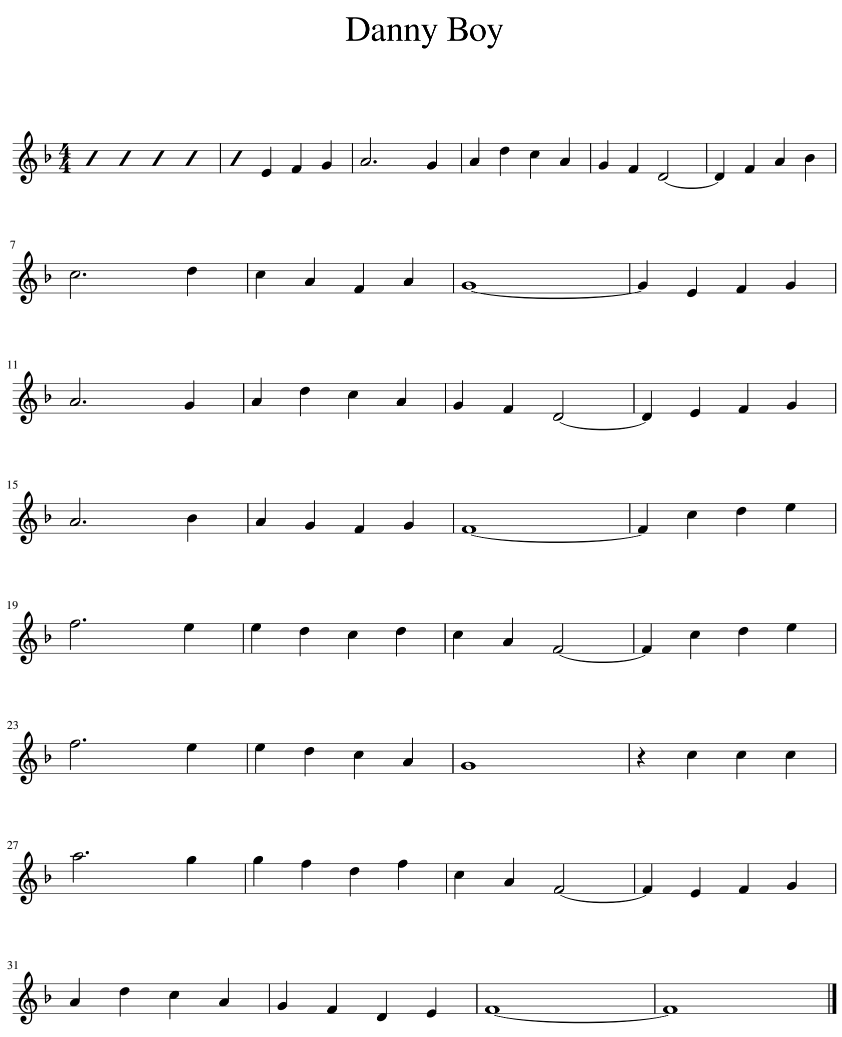

Figure S1. Sheet music for Piece 1, *Danny Boy*.

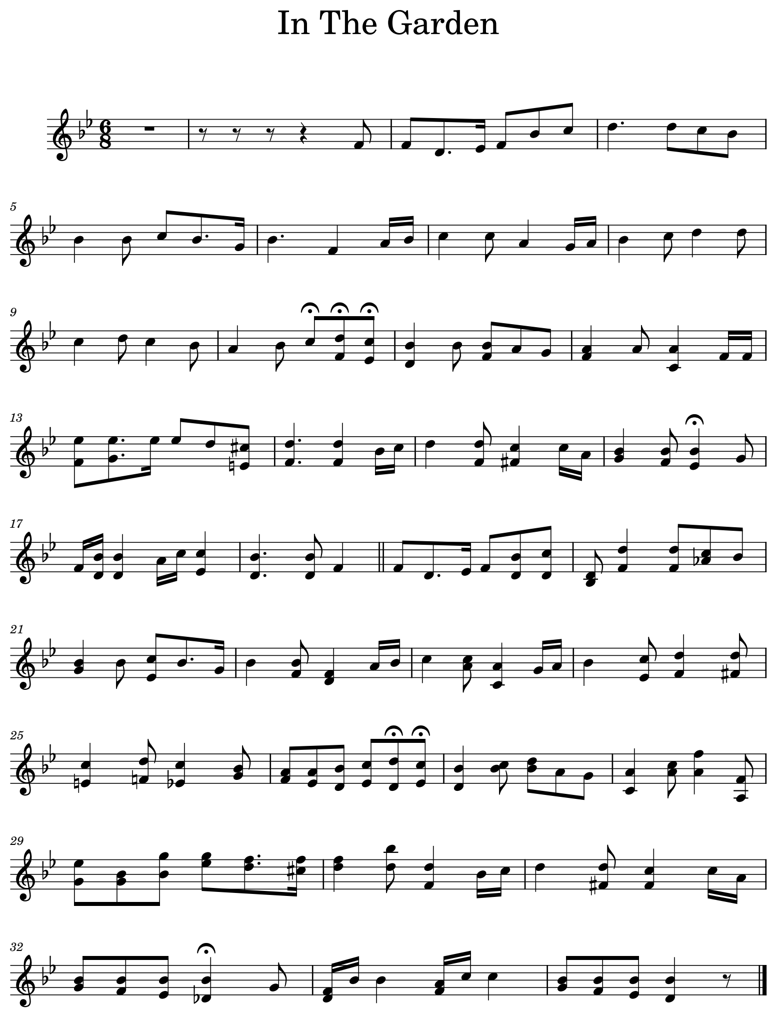

Figure S2. Sheet music for Piece 1, *In The Garden*.
